# Neoadjuvant chemoimmunotherapy achieved a pathologic complete response in stage IIIA lung adenocarcinoma harboring *RET* fusion: a case report

**DOI:** 10.3389/fimmu.2023.1258762

**Published:** 2024-01-03

**Authors:** Minqian Dai, Na Wang, Qin Xia, Yongde Liao, Wei Cao, Jun Fan, Diwei Zhou, Sihua Wang, Xiu Nie

**Affiliations:** ^1^ Department of Pathology, Union Hospital, Tongji Medical College, Huazhong University of Science and Technology, Wuhan, Hubei, China; ^2^ Department of Thoracic Surgery, Union Hospital, Tongji Medical College, Huazhong University of Science and Technology, Wuhan, Hubei, China; ^3^ Department of Nuclear Medicine, Union Hospital, Tongji Medical College, Huazhong University of Science and Technology, Wuhan, Hubei, China; ^4^ Hubei Key Laboratory of Molecular Imaging, Wuhan, Hubei, China

**Keywords:** lung adenocarcinoma, stage IIIA, RET fusion, neoadjuvant chemoimmunotherapy, pathological complete response

## Abstract

Neoadjuvant chemoimmunotherapy has demonstrated significant benefit for resectable non-small-cell lung cancer (NSCLC) excluding known *EGFR/ALK* genetic alterations. Recent evidence has shown that neoadjuvant chemoimmunotherapy could be clinically valuable in resectable localized driver gene-mutant NSCLC, though the data still lack robust support, especially for rare oncogenic mutations. Here, we report a patient with stage IIIA lung adenocarcinoma with a *RET* fusion gene and high expression of PD-L1 who underwent neoadjuvant chemoimmunotherapy and successfully attained a pathologic complete response. The patient has survived for 12 months with no recurrence or metastases after surgery. Our case suggests that this treatment strategy may be an alternative therapeutic option for resectable *RET* fusion-positive NSCLC patients.

## Introduction

*RET* fusions occur in 1–2% of NSCLCs (1). A majority of patients with *RET* -rearranged lung cancer presented stage III–IV disease at the time of initial diagnosis (2). The selective *RET* inhibitors, called selpercatinib and pralsetinib, have led to improved outcomes in the field of response rates and have shown encouraging survival rates in advanced *RET* fusion–positive NSCLCs (3, 4), promoting the recent U. S. Food and Drug Administration approval of the *RET* tyrosine kinase inhibitor (TKI) for advanced or metastatic *RET*-fusion NSCLC patients.

The clinical manifestations of resectable NSCLC patients harboring *RET* fusion include an adenocarcinoma histologic subtype, younger age, never-smoker status, solid-predominant subtype, poor differentiation, smaller diameter (≤3 cm), and early lymph node metastasis (5), which may be related to early relapse. Early operable *RET* fusion-positive NSCLC patients receive the same standard-of-care treatments as driver gene-negative NSCLC patients. These treatments include definitive locoregional therapy and/or platinum-based chemotherapy with regular follow-up (6). Increasingly, clinical trials suggest that neoadjuvant chemoimmunotherapy has promising efficacy for resectable NSCLC (7, 8). However, most neoadjuvant chemoimmunotherapy or immunotherapy clinical trials exclude patients with *EGFR/ALK* mutations, resulting in a lack of convincing evidence on the efficacy in the presence of rare driver alterations (9). The performance of neoadjuvant chemoimmunotherapy as first-line therapy across resectable NSCLCs harboring *RET* fusions has rarely been specifically reported (9, 10). Camrelizumab, a novel programmed cell death protein 1 (PD-1) inhibitor developed in China, has shown encouraging efficacy in advanced NSCLC either as monotherapy or in combination with chemotherapy (11, 12). Several recent retrospective observational studies have indicated that camrelizumab-based treatment had satisfactory efficacy for NSCLCs in neoadjuvant setting (13–15).

Herein, we present a patient with stage IIIA lung adenocarcinoma with *KIF5B-RET* fusion who responded well to neoadjuvant camrelizumab plus chemotherapy and achieved pathologic complete response (pCR).

## Case presentation

**Figure 1:**
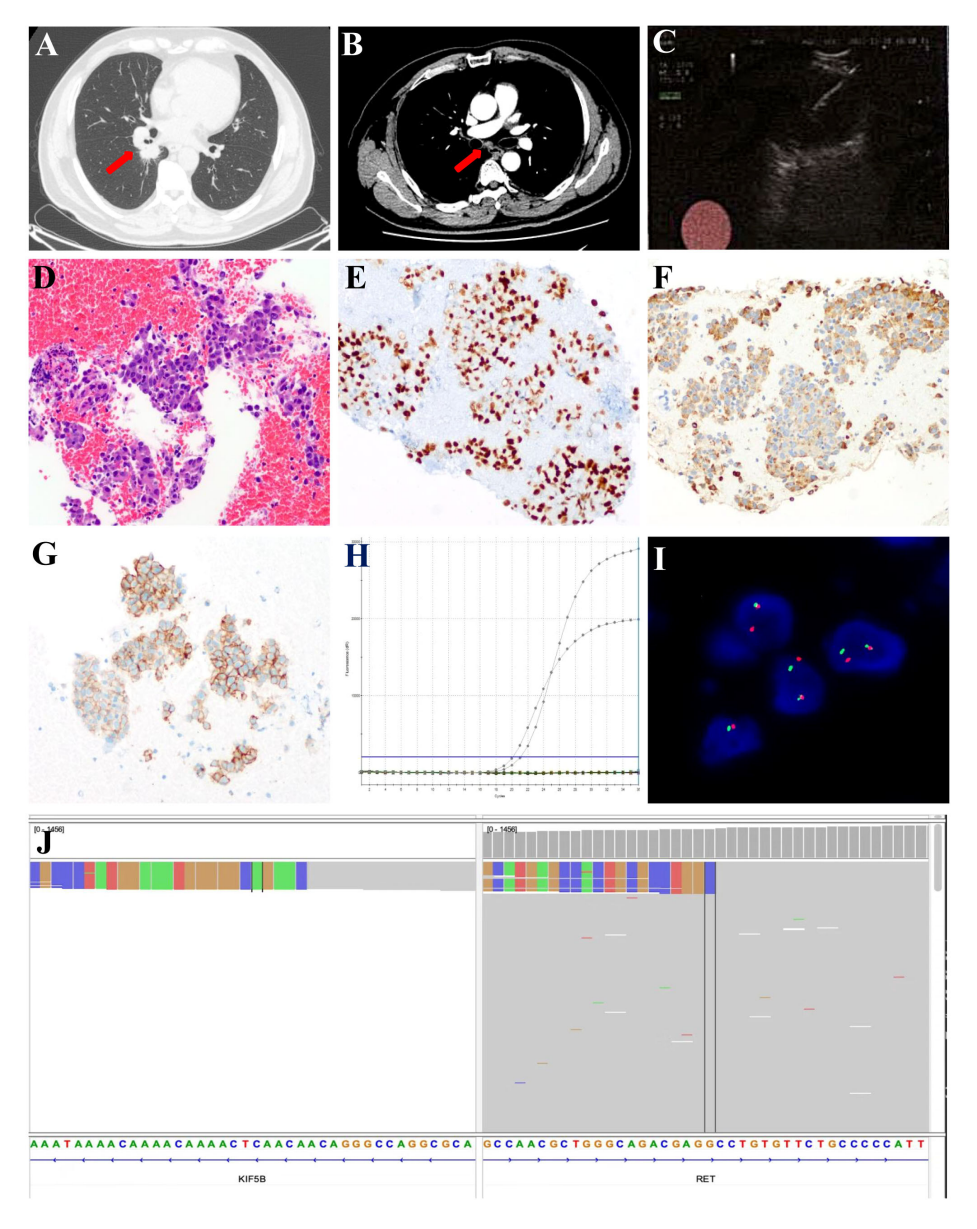
Chest radiological and pathological findings of the 7th station lymph nodes. **(A)** CT scan images of the primary tumor (red arrow) and **(B)** CT scan images of the enlarged 7th station lymph nodes (red arrow). **(C)** EBUS-TBNA for the enlarged 7th station lymph nodes. **(D)** HE-stained image of the 7th station lymph nodes biopsy specimens (Original magnification, ×200). Immunohistochemical features indicate positive expression of TTF-1 **(E)** and Napsin A **(F)**. **(G)** PD-L1 assay of the 7th station lymph nodes biopsy specimens by immunohistochemical staining (Original magnification, ×200). **(H)** The presence of *KIF5B-RET* gene fusion was identified by ARMS RT-PCR. **(I)** *RET* rearrangement was detected by the break-apart fluorescence *in situ* hybridization (FISH) assay (Original magnification, ×1000). **(J)** NGS results revealed *KIF5B-RET* gene fusion in the Integrative Genomics Viewer genome browser. ARMS, Amplification Refractory Mutation System; CT, computed tomography; EBUS-TBNA, endobronchial ultrasound with real-time guided transbronchial needle aspiration; HE, hematoxylin and eosin; PD-L1, programmed cell death-ligand 1; RT-PCR, Reverse Transcription-Polymerase Chain Reaction.

A 59-year-old Chinese male nonsmoker presented with a 1-month history of cough and discomfort in Nov 2021. The patient’s Eastern Cooperative Oncology Group (ECOG) performance status was 0. Computed tomography (CT) scan revealed a 3.2 cm × 2 cm × 3.2 cm soft tissue mass in the dorsal segment of the lower lobe of the right lung near the hilum (**Figure 1A**) and enlarged mediastinal 2R, 4R, 7th and right hilar lymph nodes (**Figure 1B**). There was no evidence of malignant lesions was observed in the central nervous system (**Supplementary Figure 1**). Endobronchial ultrasound-transbronchial needle aspiration (EBUS-TBNA) for the mediastinal lymph nodes was performed for enlarged 7th station lymph nodes (**Figure 1C**). Pathological examination showed low-differentiation metastatic lung adenocarcinoma with positive expression of TTF-1 and Napsin A (**Figures 1D–F**). Moreover, programmed cell death-ligand 1 (PD-L1) expression was found to be up to 80% in the tumor cells, as tested by the clone Dako 22C3 pharmDx antibody (**Figure 1G**). According to the 8th edition of American Joint Committee on Cancer (AJCC) staging system, the patient was clinically diagnosed with stage IIIA (cT2aN2M0) lung adenocarcinoma. The tumor tissue obtained by biopsy was evaluated by next-generation sequencing (NGS) of 68 solid tumor core genes. The sequencing assay showed that this patient had a *RET* fusion: KIF5B-*RET* (K15: R12, abundance: 2.14%) (**Figure 1J**), which was further confirmed by amplification-refractory mutation system-polymerase chain reaction technology (ADx-ARMS RT-PCR kit, Amoy Diagnostics, Xiamen, China) (**Figure 1H**) and *in situ* hybridization assay analysis with *RET* break-apart fluorescence (ZytoVision) (**Figure 1I**).

**Figure 2:**
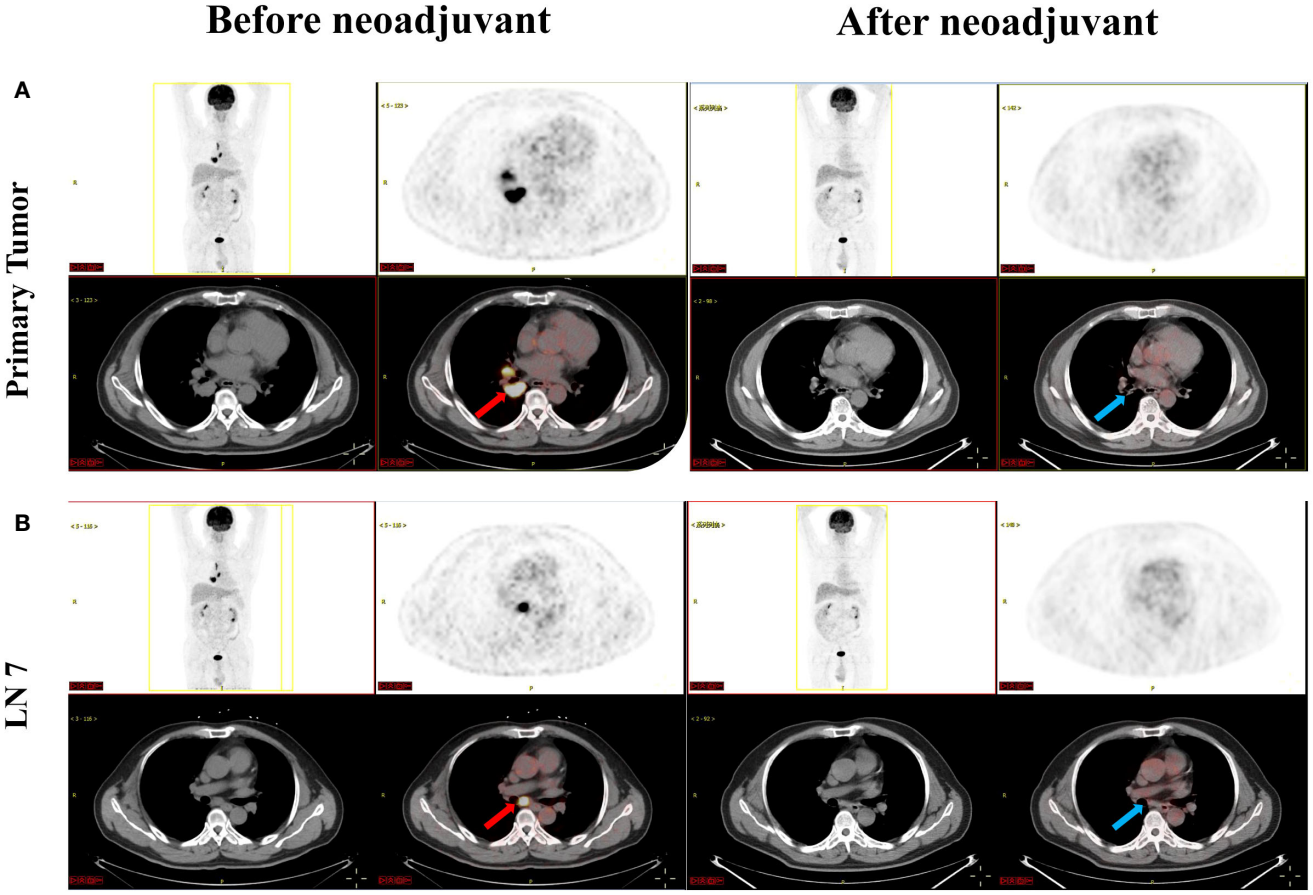
Images before (red arrow) and after (blue arrow) neoadjuvant camrelizumab plus chemotherapy treatment. **(A)** showed PET-CT of the primary tumor; **(B)** showed PET-CT of the 7th station lymph nodes. LN 7, 7th station lymph nodes; PET-CT, positron emission tomography-computed tomography.

**Figure 3:**
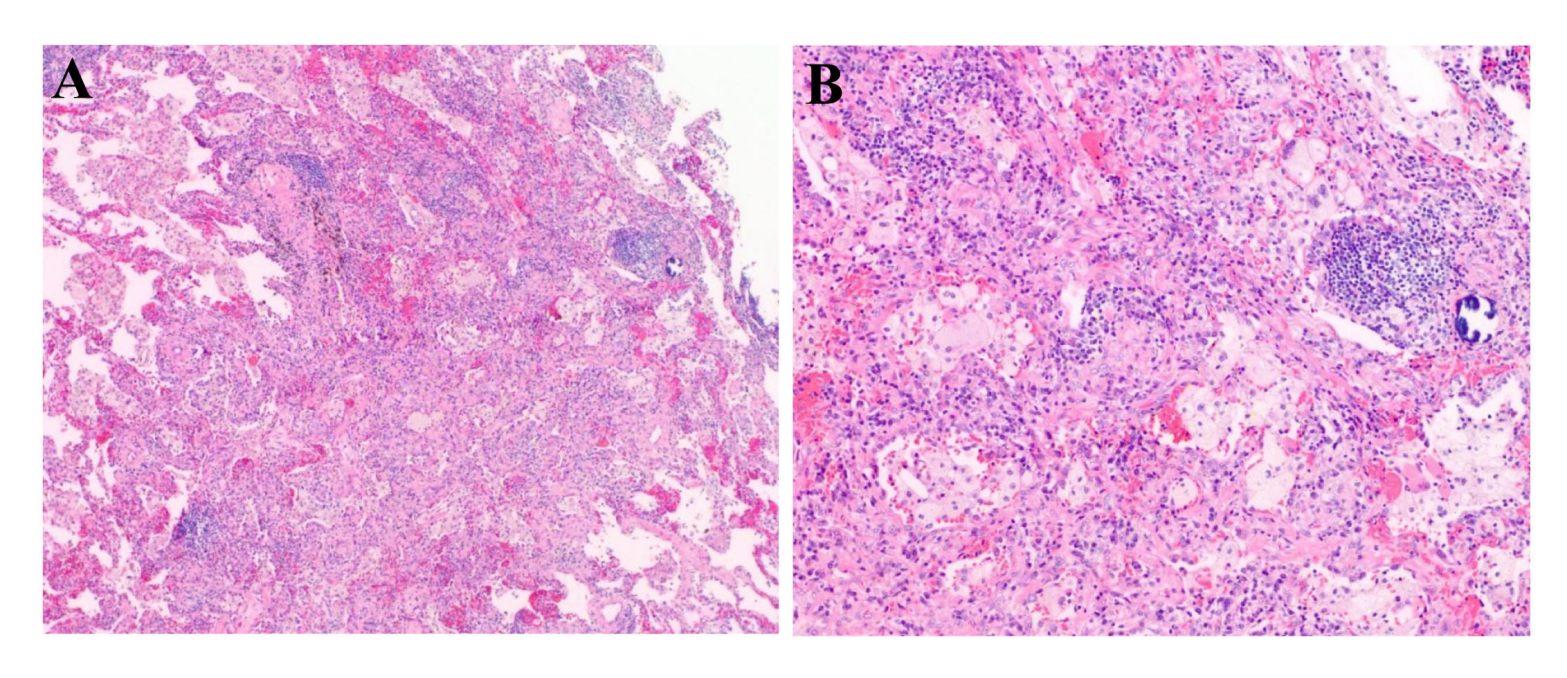
Picture of the postoperative pathological HE-staining of the primary lesion of the lower lobe of the right lung. (Original magnification, **(A)**: ×40; **(B)**: ×100). HE, hematoxylin and eosin.

**Figure 4:**
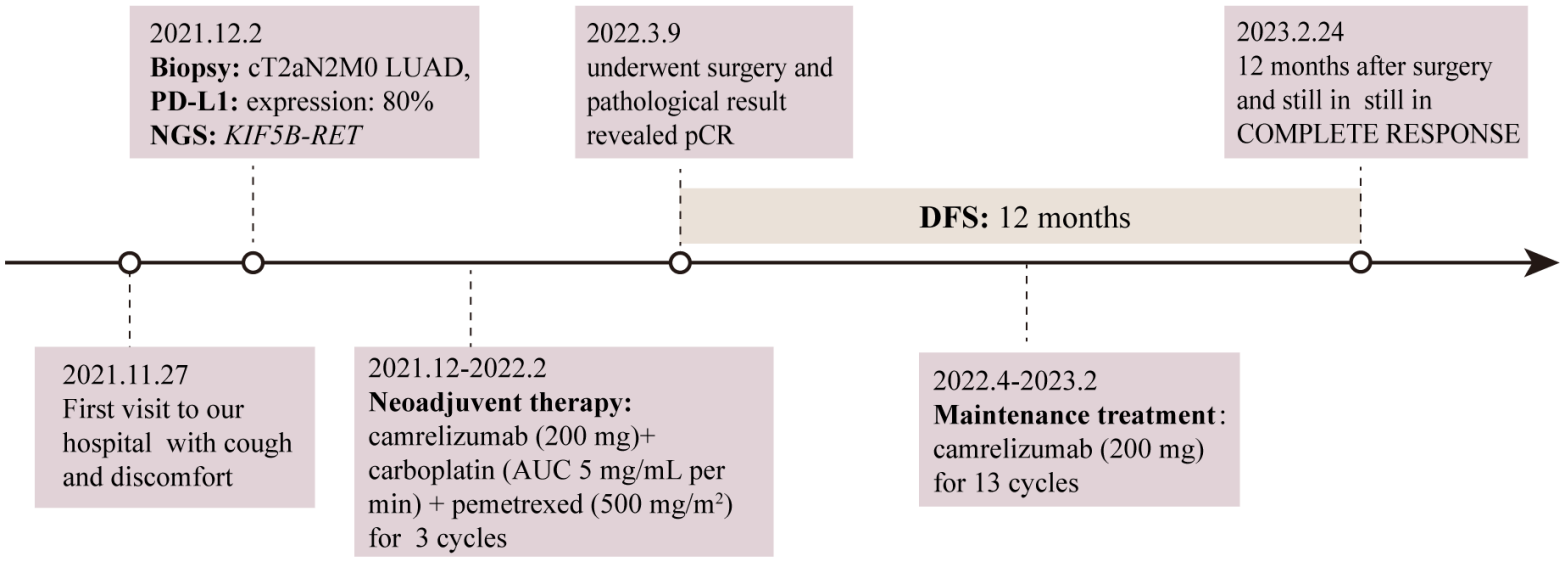
Timeline of the patient’s treatment history. LUAD, lung adenocarcinoma; PD-L1, programmed death-ligand 1; NGS, next-generation sequencing; pCR, pathological complete response; AUC, area under the curve; DFS, disease-free survival.

Currently, there is insufficient evidence regarding the neoadjuvant targeted therapy regimens for patients with *RET*-fusion resectable NSCLC. In addition, the patient could not afford highly selective *RET* inhibitors. After extensively discussing the standard regimen and the potential risks of pursuing a non-standard treatment option with the patient, a combination of chemotherapy and immunotherapy adopted after he gave informed consent. The patient was started on camrelizumab (200 mg) combined with carboplatin (area under the curve 5 mg/mL per min) plus pemetrexed (500 mg/m^2^), administered every 3 weeks for 3 cycles on 2021-12-21, 2022-01-13, and 2022-02-07. Adverse events of the drugs were well controlled and tolerable. There were no grade 4 toxicities during neoadjuvant therapy. Posttreatment radiologic evaluation by PET-CT showed that the primary lesion and mediastinal lymph nodes had decreased in size and had slowed their metabolism when compared with the disease status in Nov 2021 (**Figure 2**). The disease response was considered a partial response (PR) according to Response Evaluation Criteria in Solid Tumors (RECIST) version 1.1 after the 3rd cycles of chemoimmunotherapy. Following multidisciplinary discussion and evaluation, the patient underwent a video-assisted thoracic surgery of lower lung lobectomy plus radical mediastinal lymph node dissection (including the 2nd/4th station (3 nodes), 7th station (2 nodes), 11th station (2 nodes), 12th station (3 nodes), 13th station (1 nodes) and other paratracheal lymph nodes (6 nodes)) on 9 March 2022, which was 4 weeks after he completed neoadjuvant therapy. The postoperative pathologic examination demonstrated the absence of any remaining viable tumor cells in both the primary lesion and resected lymph nodes, thereby confirming the patient’s attainment of a pCR subsequent to neoadjuvant chemoimmunotherapy (**Figures 3A, B**). After surgery, the patient was treated with adjuvant immunotherapy using camrelizumab (200 mg/m2) for 13 cycles, and no recurrence or complications were observed during the 12-month follow-up (**Figure 4**).

All procedures performed in the human participant were in accordance with the ethical standards of the institutional and national research committee(s) and with the Helsinki Declaration (as revised in 2013). All treatments were performed with the consent of the patient. Written informed consent was obtained from the patient for publication of this study and any accompanying images.

## Discussion

Patients with *RET-*rearranged lung adenocarcinomas make up a rare and heterogeneous NSCLC subgroup (16). Selective *RET* inhibitors have demonstrated remarkable and durable success against advanced *RET* fusion-positive NSCLC, but there is less evidence in the neoadjuvant setting. More recently, studies revealed that neoadjuvant immunotherapy plus chemotherapy has had a significant benefit in driver gene-negative patients but has also indicated its potential clinical feasibility for treating resectable localized oncogene-mutant NSCLC (9). However, the data is still limited for patients possessing *RET* fusion genes, and more available data are needed to support it use. Here, we describe the beneficial effect of neoadjuvant chemoimmunotherapy in a stage IIIA NSCLC patient who harbored a *RET* fusion gene with high PD-L1 expression.

Encouragingly, the selective *RET* inhibitors, selpercatinib and pralsetinib, have shown potent and durable clinical activity, with a well-tolerated safety profile in patients with advanced *RET*-altered NSCLC (3, 4). However, resectable *RET* fusion-positive NSCLC patients treated with selective TKI adjuvant and neoadjuvant therapy have only been found in scattered case reports to date. Zhou, N. et al. reported on the efficacy of pralsetinib as neoadjuvant treatment in a patient diagnosed with stage IIIA lung adenocarcinoma harboring KIF5B-*RET* rearrangement, transforming the unresectable tumor into a resectable tumor. In their study, the postoperative pathologic results revealed tumor regression rate of 74% and no residual viable tumor cells were observed in the resected lymph nodes (17). An, L. et al. reported a KIF5B-*RET* fusion patient with stage IB lung adenocarcinoma who achieved stable disease(SD) for more than 9 months with second-line adjuvant pralsetinib treatment (18). Because high-grade evidence of *RET* selective TKIs as neoadjuvant targeted therapy from large studies is scarce and our patient could not afford the cost of treatment, we could not perform targeted therapy.

The activity of adjuvant immunotherapy in advanced NSCLC patients carrying rare driver oncogene alterations, including *RET* fusion, has not been well characterized. A few studies have suggested that immune checkpoint inhibitors (ICIs) as adjuvant therapy show modest activity in *RET*-rearranged NSCLCs. A retrospective multicenter study compared the activity of therapeutics that included multikinase inhibitors (MKIs), chemotherapy, and single-agent immunotherapy in 45 patients with locally advanced or metastatic *RET*-altered NSCLC. The findings indicated that there was no significant difference among the groups with regard to progression-free survival (PFS). Notably, the disease control rate (DCR) by ICIs was 60% (2). In addition, Guisier et al. have shown the effectiveness of ICIs as second-line treatment in nine patients with *RET* translocation. For these patients, the overall response rate (ORR) was 38% and the median progression-free survival (mPFS) was 7.6 months (95% CI, 2.3–NR) (19). The RET-MAP study, the largest cohort study on this topic reported to date, reported 52 patients with *RET-*fusion positive NSCLC treated with ICI monotherapy with a high ORR of 23%. Two patients had complete responses (CR) to single-agent immunotherapy, and the median duration of response (mDOR) was 9.4 months (20). Nevertheless, in other retrospective studies, the efficacy of immunotherapy as monotherapy in patients with advanced *RET* fusion-positive NSCLC has not been satisfactory, with ORR ranging from 0% to 7.7% and mPFS ranging from 2.1 to 3.4 months, respectively (21–23). Considering the small sample size of the above-mentioned studies, the ICI activity in *RET*-positive NSCLC remains uncertain. In a recent study on the use of ICIs in combination with chemotherapy for patients with advanced *RET* fusion-positive NSCLC, the 12 patients who received chemoimmunotherapy as first-line treatment had an ORR of 70% (7/10) (24). The results suggested that immunotherapy combined with chemotherapy may have promising efficacy in *RET* fusion-positive patients, prompting the question of whether resectable NSCLC patients with *RET* fusions could also benefit from chemoimmunotherapy.

Growing evidence has demonstrated significant efficacy and favorable safety profile of neoadjuvant chemoimmunotherapy in the treatment of resectable NSCLC (25–29). Recently released data from Checkmate-816 showed that the combination of nivolumab and chemotherapy as neoadjuvant therapy resulted in significantly longer event-free survival (EFS) and a higher pCR proportion than chemotherapy alone in resectable NSCLC patients without known *EGFR/ALK* mutations. The addition of nivolumab to neoadjuvant chemotherapy did not result in an increase in adverse events or hinder surgery (26). Based on these clinical trials, the FDA granted approval for the use of nivolumab in combination with platinum doublet chemotherapy as neoadjuvant treatment for patients with resectable NSCLC. At present, there is a scarcity of data regarding the treatment of oncogene-driven NSCLC using novel neoadjuvant modalities. Zhang, C. et al. conducted a study using a large consecutive multicenter cohort of 40 patients with oncogene-mutant NSCLC (including *EGFR*, *KRAS*, *RET* fusion, *ROS1* fusion, *ALK* fusion, *HER2* and *BRAF* insertion) treated with neoadjuvant immunotherapy. In their cohort, the ORR was 62.5%, the major pathological response (MPR) rate was 37.5%, and the pCR rate was 12.5%. Among the three patients with *RET* fusion-positive NSCLC, one individual underwent neoadjuvant immunotherapy and achieved a PR, while the remaining two patients received a combination of neoadjuvant immunotherapy and chemotherapy resulting in SD and MPR, respectively (9). These findings initially indicated that neoadjuvant immunotherapy may no longer be considered a contraindication for surgically resectable localized NSCLC with oncogene mutations.

The investigation of predictive biomarkers for chemoimmunotherapy is still ongoing. PD-L1 status, which is widely recognized as a predictive biomarker for immunotherapy in advanced NSCLC, tends to be expressed at low-to-intermediate levels in *RET-*rearranged lung cancers. Previous reports have reported that the tumor proportion score of PD-L1≥1% ranged from 42% to 77.8% and high PD-L1 expression (≥50%) was observed in 19% to 37% of tumor cells in these patients (20, 22, 30, 31). The predictive role of PD-L1 in immunotherapy for patients with advanced *RET*-rearranged lung cancer remains a subject of controversy. Offin et al. conducted a study involving 13 advanced *RET*-rearranged NSCLC patients to demonstrate the effectiveness of ICIs. The mPFS in all patients was 3.4 months (95% CI, 2.1 to 5.6 months). However, patients with higher levels of PD-L1 expression (50% and 30%) experienced shorter PFS durations of 1.3 months and 2.5 months, respectively (22). In a separate case series, Rodriguez et al. observed that patients with *RET*-arranged lung cancers and high PD-L1 expression exhibited positive responses to immunotherapy (32). Furthermore, Baby, S. et al. documented a case of a patient with metastatic *RET*-rearranged NSCLC and 100% positive PD-L1 expression who obtained a CR lasting for 29 months and ongoing, following frontline pembrolizumab treatment (33). Similarly, Nakasuka, T. et al. reported the positive impact of pembrolizumab in a patient with 90% positive PD-L1 expression advanced NSCLC harboring the CCDC6–*RET* fusion gene and NF1/TP53 mutations (34). In the present case, PD-L1 expression positivity reached 80%, which likely accounts for part of the favorable outcome of this treatment strategy. Taken together, more effective predictive biomarkers for identifying patients with oncogene-addicted NSCLC who would benefit from chemoimmunotherapy require further exploration and validation in future clinical trials.

In the present case, ICI-based regimen was applied in neoadjuvant therapy and postoperative adjuvant therapy setting, and immune-mediated toxicity was tolerable during treatment period. No RET-TKIs was used in this reported case. Additionally, the toxicity risk associated with concurrent or sequential treatment with ICIs and RET-TKIs has not been adequately explored so far and only sporadic reports involved. Gu et al. reported a case of the pralsetinib efficacy and safety in a stage IIIA NSCLC with 3 concurrent *RET* fusions (*CCDC6-RET, LINCO1264-RET* and *SEMA5A-RET*), who was first treated with multiple lines of chemotherapy and switched to ICI (toripalimab) for 2 cycles but failed to respond (35). Despite with a renal insufficient background, adverse events of pralsetinib therapy included grade 2 creatinine elevation and grade 1 proteinuria, hypertension and rash after dose reduction to 100 mg. The above case suggested that sequential RET-TKIs therapy after ICIs failure may still be effective and toxicity-manageable. Actually, ICIs-based regimens were commonly used as later lines therapy in advanced RET fusion-positive patients according to the limited evidence from real-world studies. In a retrospective, multicenter study, ICIs were used as ≥2nd-line treatment in 9 advanced RET-fusion NSCLC patients and researchers reported 10% of enrolled patients were observed grade 3 to grade 5 immune-mediated AEs (most frequent was colitis) (19). Furthermore, another RET−fusion positive case with brain metastases and disease progression after previous RET-TKIs therapy, the toxicity of subsequent ICIs-based treatment was manageable and no ICIs discontinuance for severe adverse events occurred (36). At present, related research concerning the toxicity risk associated with concurrent or sequential adiministration with ICIs and RET-TKIs is very limited and further study will be explored in the future.

## Conclusion

In summary, we provide the first direct evidence that resectable NSCLC with *RET* fusion and strongly positive PD-L1 staining (tumor proportion score = 80%) had a significant response to neoadjuvant chemoimmunotherapy, indicating that neoadjuvant chemoimmunotherapy may be a promising candidate for treating resectable *RET* fusion-positive NSCLC patients with high expression of PD-L1. Future investigations will help to clarify the optimal timing to use chemotherapy plus immunotherapy in these patients and the potential clinical feasibility in neoadjuvant setting.

## Data availability statement

The original contributions presented in the study are included in the article/**Supplementary Material**. Further inquiries can be directed to the corresponding authors.

## Ethics statement

The studies involving humans were approved by the ethics committee of Union Hospital, Tongji Medical College, Huazhong University of Science and Technology. The studies were conducted in accordance with the local legislation and institutional requirements. The participants provided their written informed consent to participate in this study. Written informed consent was obtained from the individual(s) for the publication of any potentially identifiable images or data included in this article.

## Author contributions

MD: Data curation, Investigation, Writing – original draft. NW: Data curation, Investigation, Writing – original draft. QX: Data curation, Investigation, Writing – original draft. YL: Writing – review & editing. WC: Writing – review & editing. JF: Writing – review & editing. DZ: Writing – review & editing, Conceptualization. SW: Conceptualization, Writing – review & editing. XN: Writing – review & editing, Funding acquisition, Project administration.

## Supplementary material

Supplementary Figure 1 The images of PET-CT in central nervous system. **(A)** and **(B)** showed no sign of malignant lesions was observed in the brain.
