# Supplementary figures and images for: Neoadjuvant chemoimmunotherapy achieved a pathologic complete response in stage IIIA lung adenocarcinoma harboring RET fusion: a case report
Source: Front Immunol. 2024 Jan 3;14:1258762. doi: 10.3389/fimmu.2023.1258762 (PMC10791793; doi:10.3389/fimmu.2023.1258762)

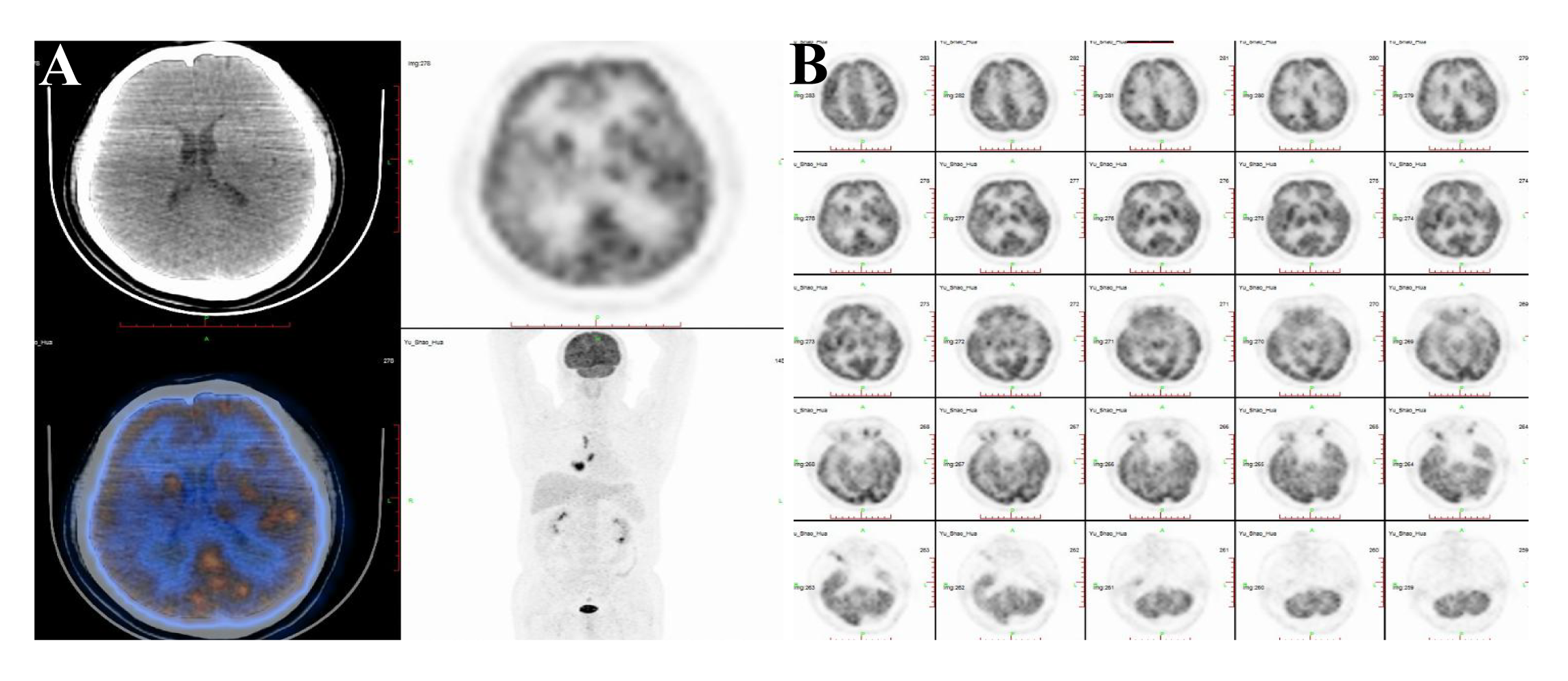

Supplement: Supplementary Figure 1 — The images of PET-CT in central nervous system. (A) and (B) showed no sign of malignant lesions was observed in the brain. [file Image_1.tif]
